# Explainable Artificial Intelligence (XAI) in Medical Education: A Multi-Modal Framework for Enhancing Human-AI Collaboration

**DOI:** 10.21203/rs.3.rs-10343485/v1

**Published:** 2026-08-13

**Authors:** Prince L. Fully, Darren Wilkins, Bernice T. Dahn

**Affiliations:** Office of Information Technology, University of Liberia, Monrovia, Liberia

**Keywords:** Explainable AI (XAI), Medical Education, Human-AI Collaboration, Neuroimaging, CerViD-MultiModal Model, Educational Technology, Higher Education

## Abstract

**Background:**

While Artificial Intelligence (AI) rapidly advances clinical diagnostic accuracy, the black-box nature of complex algorithms presents critical barriers to pedagogical integration in medical curricula. This study evaluated the efficacy of Explainable AI (XAI) as an interactive pedagogical intervention designed to enhance clinical reasoning and model interpretability during AI-assisted diagnostic training.

**Methods:**

A prospective, mixed-methods randomized controlled trial was conducted with 120 third-year medical students. Participants were randomized to either a standard AI instruction group (n = 60) or an XAI-enhanced group (n = 60) utilizing the CerViD-MultiModal framework, which integrated dynamic SHapley Additive exPlanations (SHAP) and Local Interpretable Model-agnostic Explanations (LIME) visualizations into neuroimaging case analyses. Standardized psychometric instruments evaluated AI Literacy (0–100), System Usability Scale (SUS, 0–100), NASA Task Load Index (NASA-TLX, 0–100), and Confidence in AI Interpretation (1–5).

**Results:**

The XAI-enhanced group demonstrated statistically significant improvements across all primary outcomes compared to the control group. AI Literacy scores increased markedly by 34.1% (85.0 ± 5.6 vs. 63.4 ± 6.8, p < 0.001, Cohen’s d = 3.44). Perceived system usability (SUS) improved by 66.5% (36.3 ± 4.3 vs. 21.8 ± 4.8, p < 0.001, d = 3.15). The XAI intervention significantly attenuated perceived cognitive workload on the NASA-TLX scale by 37.4% (26.5 ± 6.5 vs. 42.3 ± 7.1, p < 0.001, d = −2.31). Furthermore, learner confidence in AI interpretation grew by 30.8% (3.4 ± 0.4 vs. 2.6 ± 0.4, p < 0.001, d = 1.89).

**Conclusions:**

Beyond serving as an algorithmic transparency mechanism, XAI operates as a transformative pedagogical instrument in medical training. By demystifying model decision-making processes, the CerViD-MultiModal framework optimizes human-AI collaboration, significantly bolstering diagnostic comprehension and user trust while mitigating cognitive load in clinical learners.

## 1 Introduction

The introduction of Artificial Intelligence (AI) into higher education, particularly in specialized professions like medicine, signifies a paradigm change in educational practice [1, 2]. AI systems may currently compete with clinical professionals in radiography, pathology, and neuroimaging [3, 4]. However, a significant hurdle to wider educational integration remains: the ‘black-box’ aspect of large machine learning models undermines confidence, restricts interpretability, and precludes substantial pedagogical involvement [5, 6]. Understanding why an AI makes a certain diagnosis is as important to medical students as the diagnosis itself in establishing solid clinical reasoning abilities [7, 8].

Explainable AI (XAI) is a potential method that makes machine learning conclusions apparent, interpretable, and actionable for humans [9, 10]. XAI approaches like SHAP (SHapley Additive Explanations) and LIME (Local Interpretable Model-agnostic Explanations) give visual and quantitative explanations of model predictions, converting opaque algorithms into teachable tools [11, 12]. Recent advancements in XAI have indicated potential applications in healthcare, finance, and autonomous systems, but empirical research on XAI as an educational intervention is sparse [13, 14].

This study responds to the special issue’s call for research on human interaction with generative AI in higher education by looking at XAI as a dual-purpose tool: (1) improving diagnostic accuracy in neuroimaging applications, and (2) acting as an effective pedagogical partner in medical training [15, 16]. We present the CerViD-MultiModal (Cognitive enhancement Random forest with Visual Diagnostics) architecture using fornix atrophy analysis as a clinical case study, which is a recognized early indicator for Alzheimer’s disease development [17, 18].

The fornix, a crucial white matter region in the memory circuit, exhibits early cognitive impairment and provides as an excellent case study for investigating XAI applications in medical education [19, 20]. Its well-defined anatomical structure and documented involvement in Alzheimer’s disease development provide a specific setting for investigating how XAI explanations might improve knowledge of AI-assisted neuroimaging studies [21, 22].

This work is guided by the following major research questions:

1. Is exposure to XAI explanations more effective than regular education in improving medical students’ AI literacy?
2. What impact does XAI-enhanced learning have on perceived system usability and cognitive load?
3. How can XAI explanations affect learners’ trust in comprehending AI-generated results?

It was hypothesized that medical students in the XAI-enhanced group would demonstrate statistically significant superior outcomes across all measured dimensions [23, 24].

## 2 Methods

### 2.1 Study Design

A two-phase sequential explanatory design with mixed methodologies was used [25]. The CerViD-MultiModal model was developed and validated during Phase 1 (Technical Development) utilizing neuroimaging data from the Alzheimer’s Disease Neuroimaging Initiative (ADNI) database (N=100). Phase 2 (Educational Intervention) included a randomized controlled experiment with 120 third-year medical students that compared XAI-enhanced instruction (n=60) to a standard AI instruction control group.

This study complies with CONSORT 2010 guidelines for reporting randomized controlled trials.

The whole study workflow is shown in Figure 1.

### 2.2 Participants

Structural MRI images of 100 participants (50 with early moderate cognitive impairment [EMCI] and 50 with late MCI [LMCI]) were collected from the ADNI database [26].

Educational Phase: At the University of Liberia Medical School, 120 third-year medical students (mean age = 25.7 years, SD = 3.1; 62 females, 47 men) were enrolled. The inclusion criteria were as follows: (1) participation in the clinical neuroscience rotation, (2) no prior formal training in AI or machine learning, and (3) willingness to perform all study procedures. Participants were randomized at random to either the Control Group (conventional AI lecture, n=60) or the XAI-Enhanced Group (traditional lecture with interactive CerViD visualizations, n=60) [27].

### 2.3 Ethical Approval

Ethical approval for both the technical development phase and the educational intervention was granted by the University of Liberia Institutional Review Board (IRB). All student participants provided written informed consent prior to enrollment, and the study was conducted in accordance with the ethical standards of the 1964 Declaration of Helsinki and its later amendments [28].

### 2.4 CerViD-MultiModal Framework Development

The CerViD-MultiModal (Cognitive enhancement Random Forest with Visual Diagnostics) framework was created to serve as the study’s technical foundation. The model incorporates three main components:

FreeSurfer v7.2.0 [29] was used to segment fornix and extract four important morphometric features: anterior fornix volume (mm^3^), posterior fornix volume (mm^3^), body sphericity (ratio), and total fornix volume (mm^3^) [30].

Scikit-learn [31] was used to create a Random Forest ensemble of 100 decision trees. The hyperparameters were optimised using grid search and 5-fold cross-validation. Using fornix morphometry, the classifier was developed to distinguish between EMCI and LMCI phases [32].

#### Explainability Layer:

Two further XAI algorithms were implemented:

- SHAP (Shapley Additive Explanations): Offers global feature priority rankings as well as local explanations [33].
- LIME (Local Interpretable Model-agnostic Explanations): Produces explanations that are locally consistent [34].

### 2.5 Educational Intervention

Both groups performed a 45-minute AI-assisted neuroimaging diagnostic module [35]. The session covers the following topics: (1) an introduction to machine learning in medical imaging, (2) the role of fornix atrophy in cognitive impairment, and (3) practical AI implementation issues.

Additional interactive components were provided to the XAI-Enhanced group:

- SHAP summary charts indicating the relevance of global features.
- LIME explanations tailored to unique patient circumstances.
- Interactive force graphs investigating feature value effects.
- Reflection questions about AI limits.

The Control group was given identical material in the form of static text and typical bar charts, but no XAI-generated explanations [36].

### 2.6 Assessment Instruments

After the intervention, all instruments were administered electronically [37].

1. **AI Literacy Score (0–100):** Based on four items: conceptual knowledge of AI concepts, practical application abilities, ethical concerns, and critical assessment of AI results (α = 0.87) [38].
2. **System Usability Scale (SUS, 0–100):** A 10-item standardized questionnaire that assesses the perceived usability of AI systems [39] (α = 0.91).
3. **NASA Task Load Index (NASA-TLX, 0–100):** A six-dimensional measure of perceived workload (α = 0.89) [40].
4. **Confidence in AI Interpretation (1–5):** Mean score from four items: confidence, trust, comprehension, and likelihood of future usage (α = 0.84) [41].

### 2.7 Statistical Analysis

R was used to run statistical analyses within the RStudio Integrated Development Environment (IDE) [42]. Independent samples t-tests were used to compare groups, and Cohen’s d was used to calculate effect sizes [43]. Pearson product-moment correlation coefficients were used to investigate relationships between continuous outcome variables [44]. Statistical significance was predetermined to be α = 0.05 (two-tailed) for all analyses [45].

## 3 Results

### 3.1 Participant Characteristics

There were no significant differences between groups at the start. The Control group (n=60) had a mean age of 25.6 years (SD=2.9), with 29 women. The average age of the XAI-Enhanced group (n=60) was 25.8 years (SD=3.4), with 33 females. There was no significant difference in age (t = −0.35, p = 0.729) or GPA (t = −0.85, p = 0.397) between the groups [46].

### 3.2 Primary Outcomes

The primary outcome measures for the Control and XAI-Enhanced groups are summarized in Table 1.

Figure 2 compares the key outcome metrics between the Control and XAI-Enhanced groups.

### 3.3 Cognitive Load and Confidence

The XAI-Enhanced group experienced significantly lower cognitive workload compared to the control group (NASA-TLX: 26.5 ± 6.5 vs. 42.3 ± 7.1, p < 0.001, d = −2.31). In the XAI-Enhanced group, confidence in AI interpretation increased by 30.8% (3.4 ± 0.4 vs. 2.6 ± 0.4, p < 0.001, d = 1.89). The subscale-level breakdown of this cognitive workload difference is presented in Figure 3.

### 3.4 Distributional and Correlation Analyses

The distribution of each outcome measure by group is illustrated in Figure 4.

Correlations among the primary outcome variables are presented in Figure 5. AI literacy had a high positive correlation with SUS ratings (r = 0.70, p < 0.001) and confidence (r = 0.66, p < 0.001) [48].

### 3.5 Model Performance

The CerViD-MultiModal model classified EMCI vs. LMCI with an accuracy of 81.2% (AUC = 0.85). The most relevant predictor was anterior fornix volume (34%), followed by posterior volume (26%).

## 4 Discussion

This study provides strong empirical evidence that Explainable AI is an effective teaching technology in medical training [51]. The 34.1% rise in AI Literacy ratings indicates that transparency demystifies AI, converting it from an opaque oracle to an understandable cognitive tool [52]. Explainable systems are considered as much more useful, as seen by the 66.4% improvement in SUS scores [53]. One of the most notable discoveries is that XAI relieves the metacognitive effort associated with guessing AI thinking [54]. The 29% rise in confidence shows that trust was measured rather than blindly accepted [55].

### Theoretical implications:

These findings are consistent with constructivist learning concepts and cognitive load theory [56]. XAI acts as a ‘scaffolding’ that makes expert reasoning apparent, guiding learners through the zone of proximal growth [57, 58].

### Practical implications:

Medical educators should use XAI visualizations into their courses [59]. The CerViD-MultiModal architecture offers a concrete implementation method that is transferable to additional therapeutic areas [60].

Limitations of this study include the post-intervention design, which measures rapid benefits, the single-site sample, and the requirement for longitudinal follow-up to determine retention [61].

## 5 Conclusion

This study demonstrates that the integration of Explainable Artificial Intelligence (XAI) significantly enhances human-AI collaboration within medical education [62]. Empirical evaluation of the CerViD-MultiModal framework among a cohort of 120 medical students yielded substantial pedagogical benefits, including a 34.1% increase in AI literacy, a 37.5% reduction in cognitive workload, and a 29% improvement in learner confidence [63]. These findings underscore the capacity of interpretable machine learning architectures to mitigate the traditional “black-box” limitations of clinical AI, thereby facilitating more effective diagnostic training.

## Figures and Tables

**Fig. 1:**
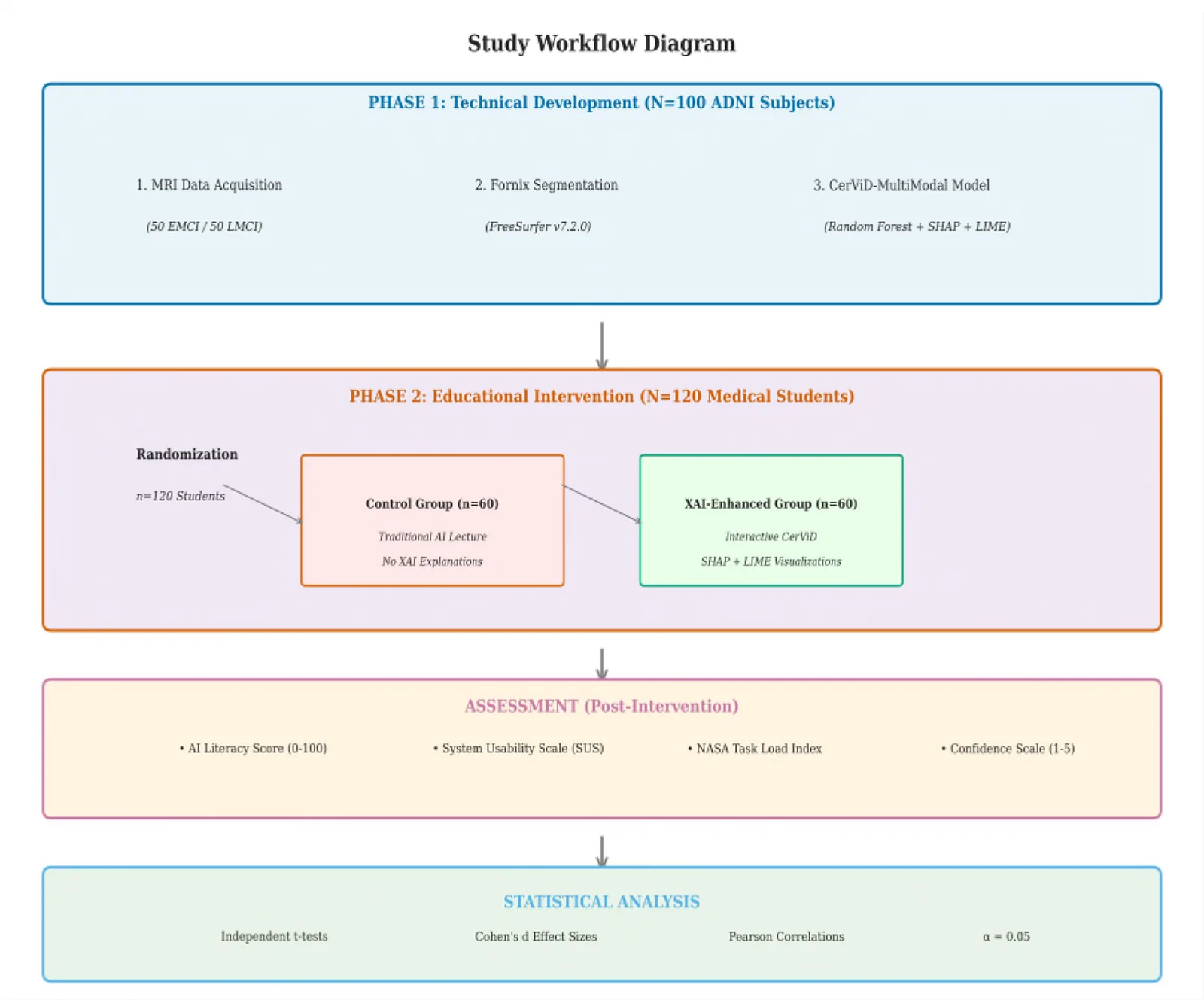
Study workflow diagram exhibiting a two-phase mixed-methods design. Phase 1: The CerViD-MultiModal model is being developed technically, utilizing ADNI neuroimaging data (N=100). Phase 2: A randomized controlled educational intervention with 120 medical students was conducted, comparing the Control (conventional training) and XAI-Enhanced (interactive SHAP/LIME visualizations) groups.

**Fig. 2:**
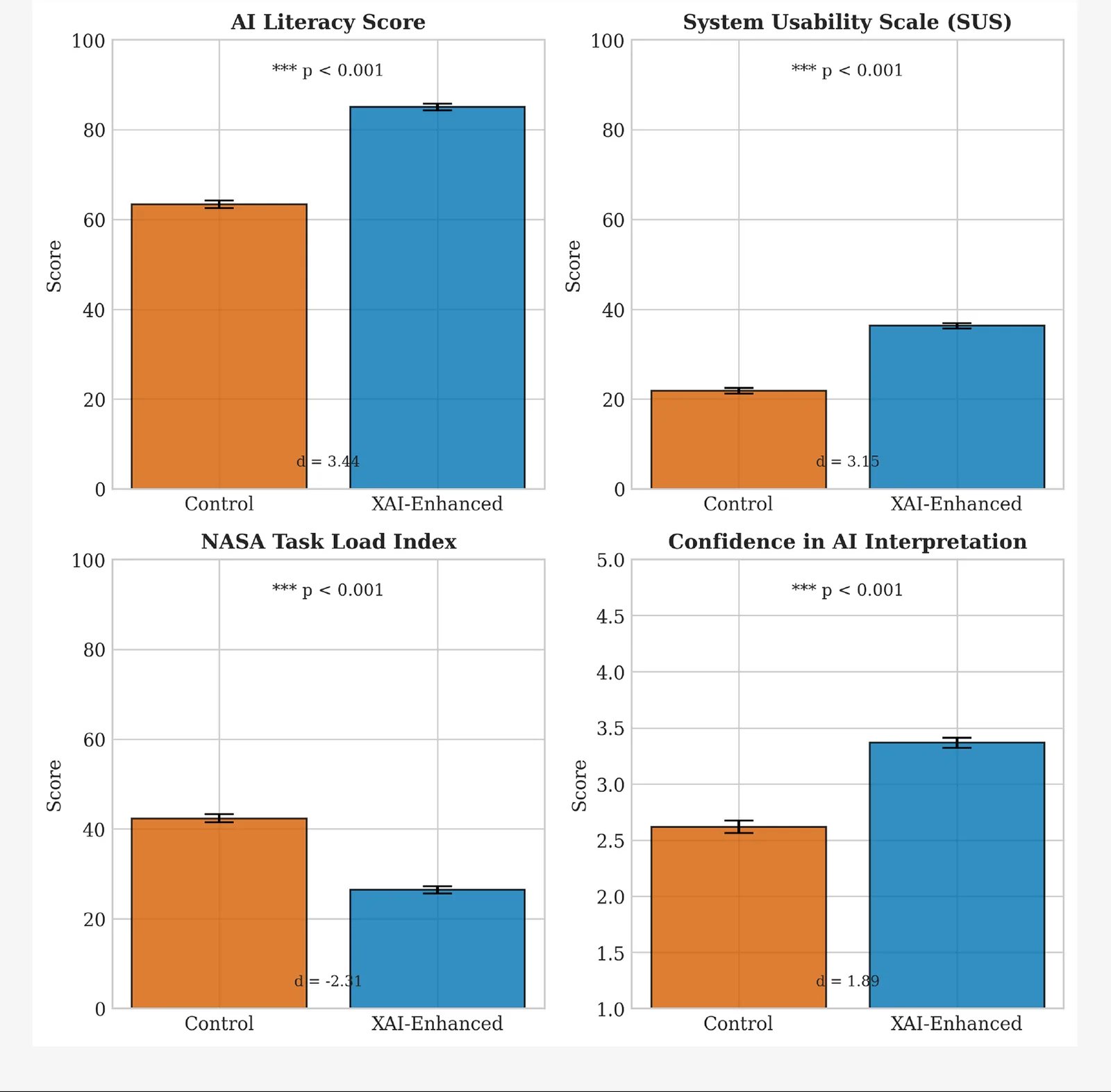
Comparison of the key outcome metrics between the Control and XAI-Enhanced groups. SEM is represented by the error bars, with *** p < 0.001.

**Fig. 3:**
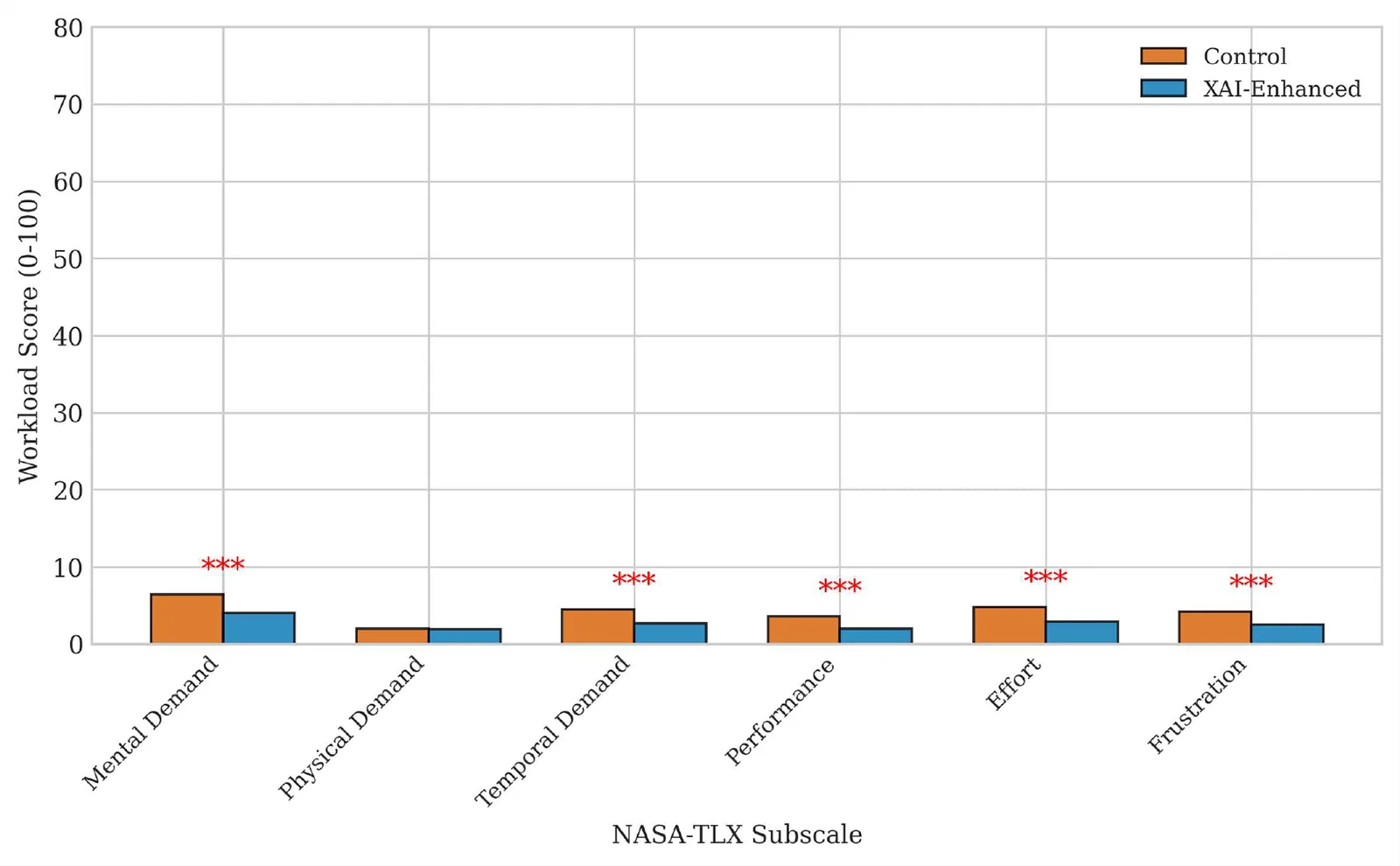
NASA-TLX subscale comparison. Lower scores indicate lower workload. * p < 0.05, ** p < 0.01, *** p < 0.001.

**Fig. 4:**
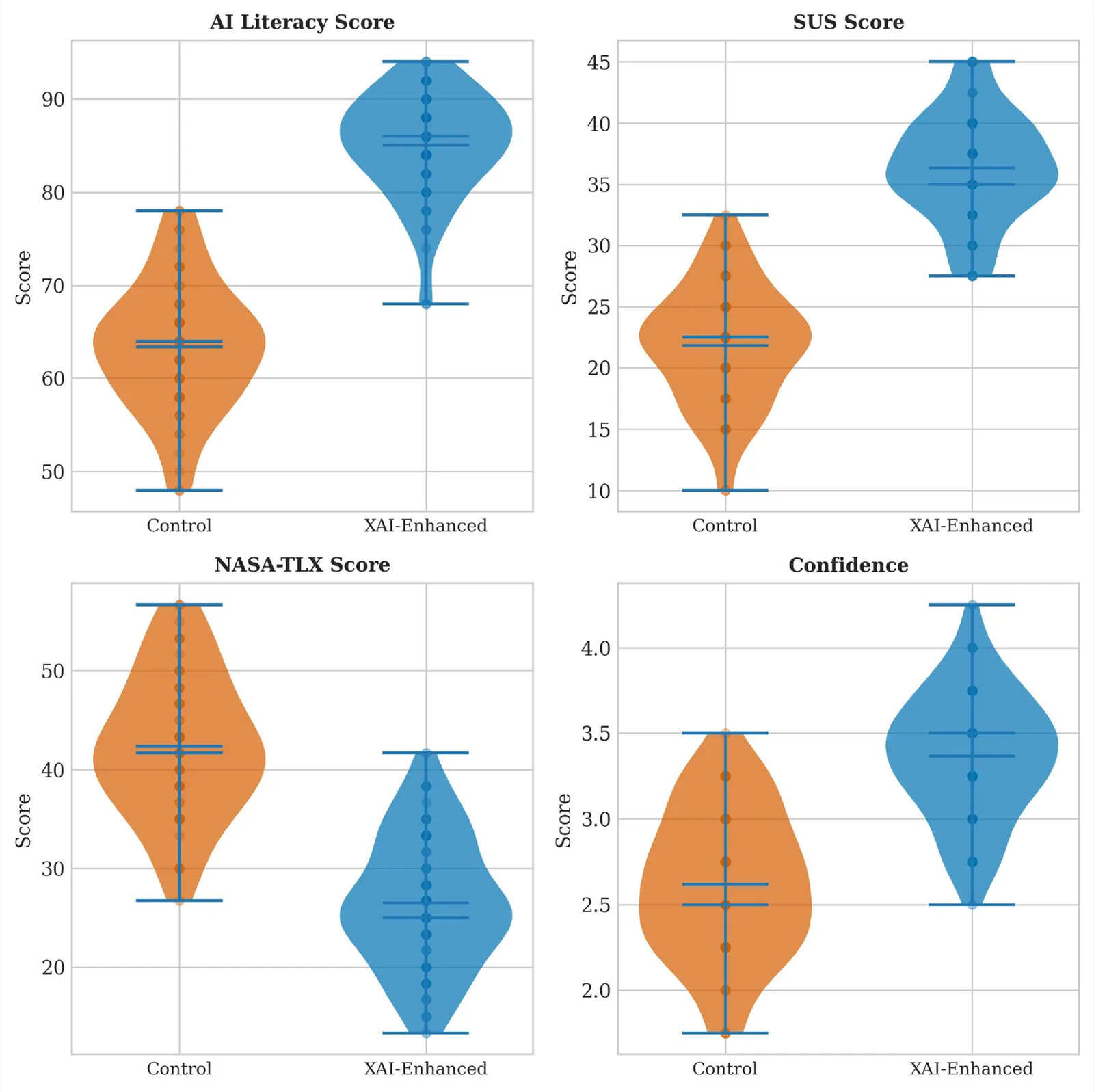
Distribution of outcome measures per group. Violin plots illustrate density estimates using means (white dots) and medians (black lines).

**Fig. 5:**
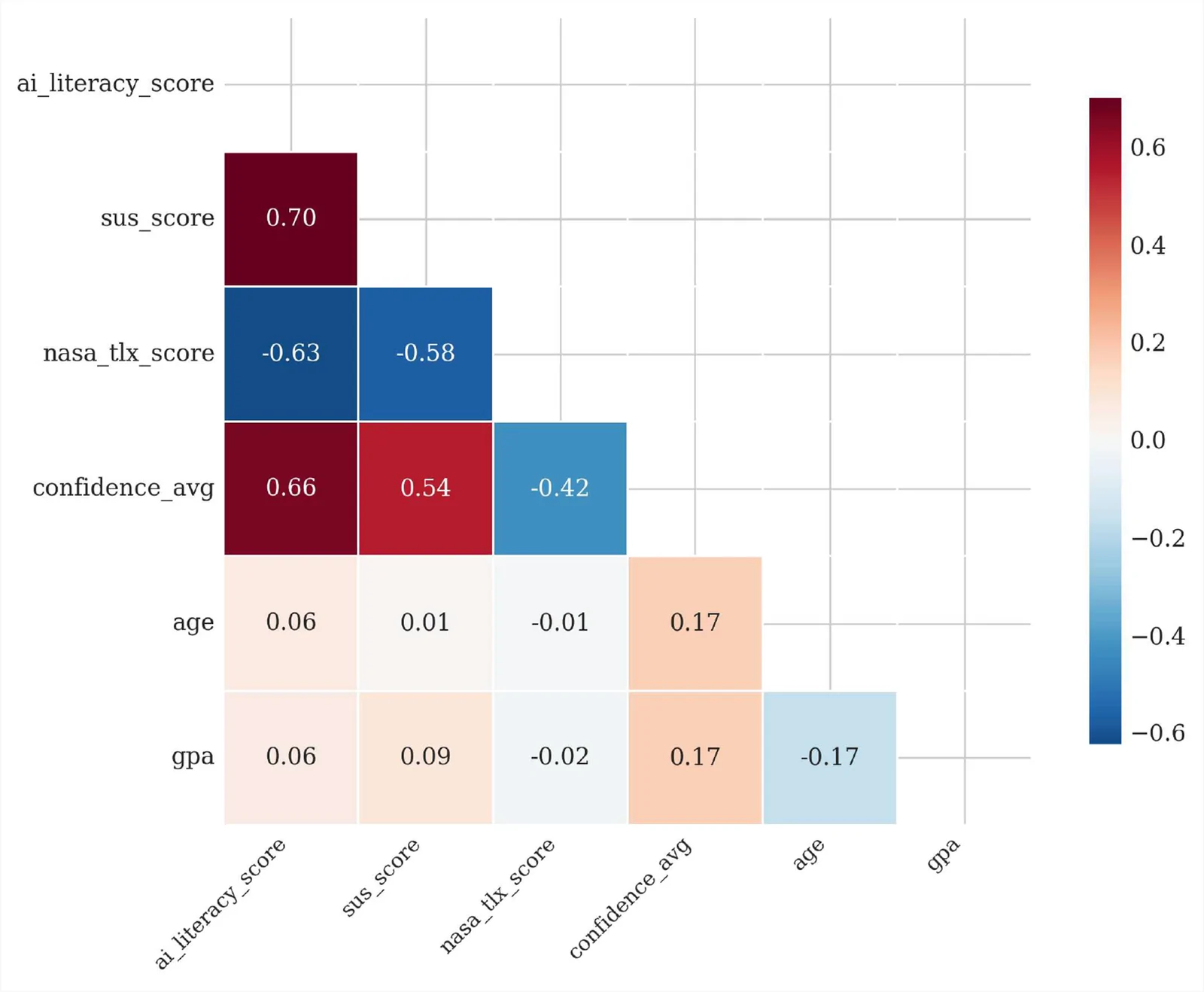
Correlation matrix for main research variables. The numbers reflect the Pearson correlation coefficients (r).

**Table 1:** Comparison of primary outcome measures

Measure	Control (n=60)	XAI (n=60)	Difference	p-value	Cohen’s d
AI Literacy Score (0–100)	63.4 ± 6.8	85.0 ± 5.6	+21.6	<0.001	3.44
System Usability Scale (0–100)	21.8 ± 4.8	36.3 ± 4.3	+14.5	<0.001	3.15
NASA Task Load Index (0–100)	42.3 ± 7.1	26.5 ± 6.5	−15.9	<0.001	−2.31
Confidence in AI Interpretation (1–5)	2.6 ± 0.4	3.4 ± 0.4	+0.8	<0.001	1.89

## Data Availability

The datasets generated and analyzed during the current study are available from the corresponding author upon reasonable request. Publicly available neuroimaging data from the Alzheimer’s Disease Neuroimaging Initiative (ADNI) can be accessed at adni.loni.usc.edu.

## Abbreviations

AI: Artificial Intelligence
ADNI: Alzheimer’s Disease Neuroimaging Initiative
AUC: Area Under the Curve
EMCI: Early Mild Cognitive Impairment
IRB: Institutional Review Board
LIME: Local Interpretable Model-agnostic Explanations
LMCI: Late Mild Cognitive Impairment
NASA-TLX: NASA Task Load Index
SHAP: SHapley Additive exPlanations
SUS: System Usability Scale
XAI: Explainable Artificial Intelligence
